# Atypical Association of Angelman Syndrome and Klinefelter Syndrome in a Boy with 47,XXY Karyotype and Deletion 15q11.2-q13

**DOI:** 10.1155/2014/517091

**Published:** 2014-10-14

**Authors:** Javier Sánchez, Ana Peciña, Olga Alonso-Luengo, Antonio González-Meneses, Rocío Vázquez, Guillermo Antiñolo, Salud Borrego

**Affiliations:** ^1^Department of Genetics, Reproduction and Fetal Medicine, Institute of Biomedicine of Seville (IBIS), University Hospital Virgen del Rocío/CSIC/University of Seville, 41013 Seville, Spain; ^2^Centre of Biomedical Network Research on Rare Diseases (CIBERER), 41013 Seville, Spain; ^3^Department of Pediatrics, University Hospital Virgen del Rocío, Avenida Manuel Siurot s/n, 41013 Seville, Spain; ^4^Department of Neurophysiology, University Hospital Virgen del Rocío, Avenida Manuel Siurot s/n, 41013 Seville, Spain

## Abstract

Angelman syndrome (AS, OMIM 105830) is a neurogenetic disorder with firm clinical diagnostic guidelines, characterized by severe developmental delay and speech impairment, balanced and behavioral disturbance as well as microcephaly, seizures, and a characteristic electroencephalogram (EEG). The majority of AS cases (70%) are caused by a 15q11.2-q13 deletion on the maternally derived chromosome. The frequency of AS has been estimated to be between 1/10000 and 1/20000. Klinefelter syndrome (KS) occurs due to the presence of an extra X chromosome (karyotype 47,XXY). The main features in KS are small testes, hypergonadotropic hypogonadism, gynecomastia, learning difficulties, and infertility. We present what is, to our knowledge, the first case of a patient with both KS and AS due to a 15q11.2-q13 deletion on the maternally derived chromosome and an extra X chromosome of paternal origin. He showed dysmorphic features, axial hypotonia, and delayed acquisition of motor skills. Early diagnosis is essential for optimal treatment of AS children; this is one of the earliest diagnosed cases of AS probably due to the presence of two syndromes. Clinical findings in this patient here described may be helpful to identify any other cases and to evaluate recurrence risks in these families.

## 1. Introduction

Angelman syndrome (AS, OMIM 105830) is a neurogenetic disorder with firm clinical diagnostic guidelines, characterized by severe developmental delay and speech impairment as well as balanced and behavioral disturbance. Other frequent clinical features include microcephaly, seizures, and a characteristic electroencephalogram (EEG) [1]. The majority of AS cases (70%) are caused by a 15q11.2-q13 deletion on the maternally derived chromosome. Other less frequent genetic mechanisms are paternal uniparental disomy of 15 chromosome (7%), imprinting defects (3%), or mutations in the maternal copy of the* UBE3A* gene (11%) [2]. The frequency of AS has been estimated to be between 1/10000 and 1/20000 [3]. Recurrence risk varies from <1% for deletion cases where no chromosome rearrangements or germinal mosaicism is present [4–6] to 50% for maternally inherited imprinting center deletions or UBE3A mutations [7].

Klinefelter syndrome (KS) also shows a clear clinical pattern, although no firm guidelines for diagnosis exist. The main features in KS are small testes, hypergonadotropic hypogonadism, gynecomastia, learning difficulties, and infertility [8]. KS occurs due to the presence of an extra X chromosome (karyotype 47,XXY) and the underlying putative genetic cause is that some genes escape inactivation of the extra X chromosome [9]. KS affects one in 150 per 100,000 male newborns and is the most common sex chromosome disorder in males. Since KS patients show great phenotypic variability, the majority of patients are diagnosed during the second decade of life and it is difficult to diagnose KS without cytogenetic analysis. In some instances chromosomal analysis is performed due to development delay, learning difficulties, or behavior problems [10].

Taking into account the incidence rates for both AS and KS, the anticipated incidence of both syndromes occurring together would be around 1 in 6–12 million by chance alone.

Here we present what is, to our knowledge, the first case of a patient with both KS and AS due to a 15q11.2-q13 deletion on the maternally derived chromosome. To date, few patients with KS and other microdeletion syndromes have been reported: six cases of KS and Prader-Willi syndrome (PWS) due to a 15q11.2-q13 deletion on the paternally derived chromosome have been published [11], a patient with KS and 22q11 microdeletion [12] and a combination of KS and 7q11.23 deletion (Williams syndrome) [13].

## 2. Patient Description

The propositus was the second child born to a healthy 33-year-old mother with a previous healthy son, now three years old. The parents were not consanguineous, and no remarkable family history was recorded. During pregnancy, she developed diabetes mellitus, which was well controlled by her diet. The mother reported the first fetal movements at 22-week gestation, with reduced fetal movements throughout the pregnancy. The propositus was delivered spontaneously at 37-week gestation. His birth weight was 3,190 g and head circumference was 34 cm. Apgar scores were 9 (1 min), 9 (5 min), and 10 (10 min). Severe hypotonia, feeding difficulties, and continuous crying were noted at birth.

**Figure 1:**
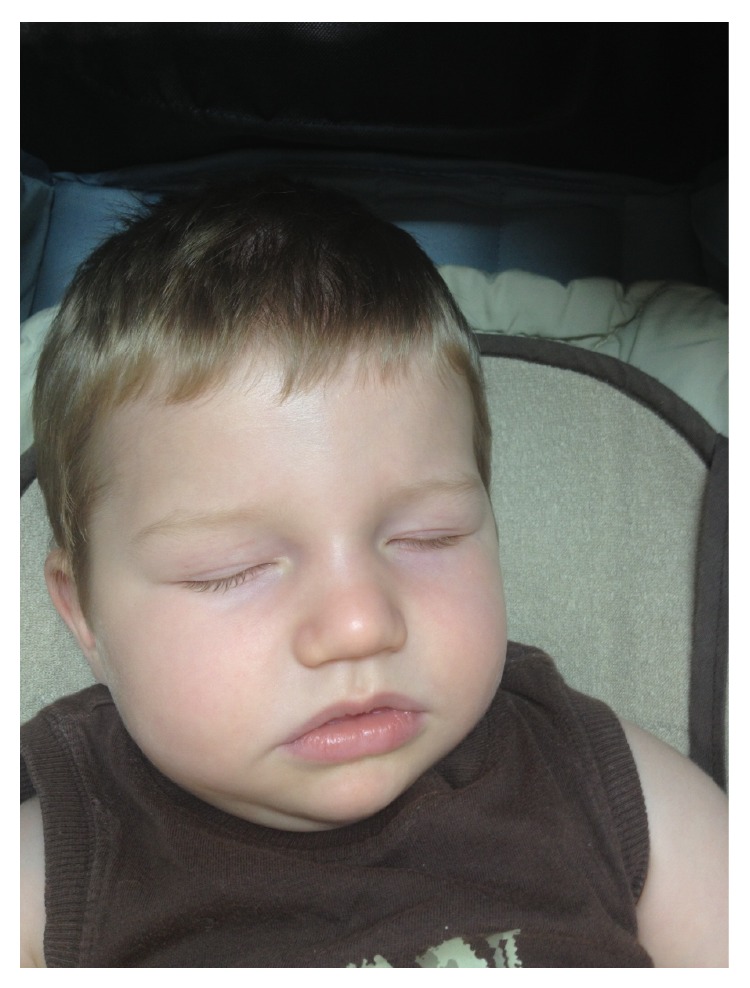
Facial appearance. Dysmorphic features, broad nasal root, and thin upper lip.

At 8 months his head circumference was 46 cm (p75), weight 9,555 g (p90), and height 70 cm (p75–p90). He showed delayed acquisition of motor skills, he could not remain seated or handle objects, and axial hypotonia was observed. He showed dysmorphic features, occipital flattening, a thin upper lip, a wide mouth, tongue protrusion, a broad nasal root, and divergent strabismus (Figure 1). A brain MRI showed a structurally normal brain.

The EEG at 8 months showed diffuse high-amplitude 4–6 Hz activity and posterior intermittent rhythmic delta waves. No epileptiform discharges were observed. Three months later, the EEG showed no significant changes. Chromosome analysis and PWS/AS were performed due to dysmorphic features and hypotonia.

Informed consent was obtained from all participants for clinical and molecular genetic studies. The study conformed to the tenets of the declaration of Helsinki as well as the requirements established by our institutional review board.

**Figure 2:**
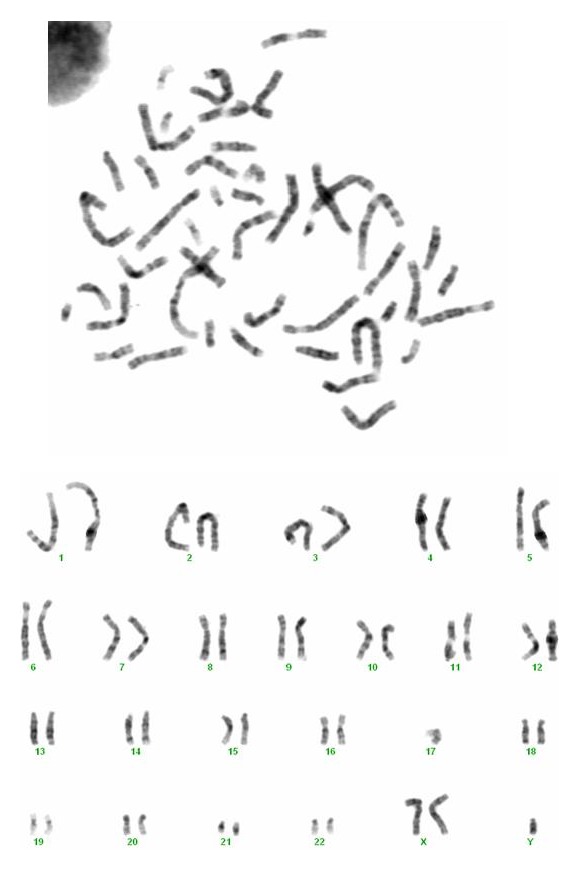
Karyotype showing the presence of an extra X chromosome.

**Figure 3:**
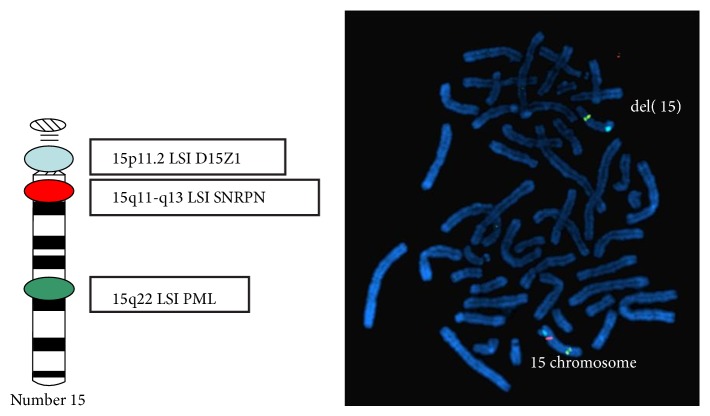
FISH analysis in metaphase with specific probes for 15 chromosome. Upper 15 chromosome with SNRPN deletion (del 15). D15Z1, SpectrumAqua; SNRPN, SpectrumOrange; PML, SpectrumGreen.

Peripheral blood cytogenetic analysis revealed 47 chromosomes, with an extra X chromosome, karyotype 47,XXY (KS). FISH analysis with specific probes (*D15Z1/SNRPN/PML*) [Vysis, Downers Grove, IL] revealed a deletion of the* SNRPN* locus. Karyotype: 47,XXY.ish del (15)(q11.2q11.2)(*SNRPN*-)[20] (Figures 2 and 3).

No deletion or rearrangements were observed in the parents' karyotypes.

**Figure 4:**
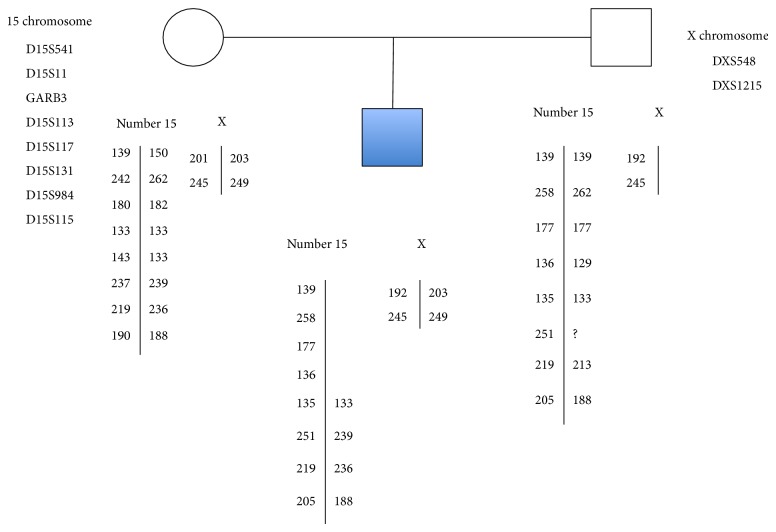
Microsatellite analysis and pedigree of the family. Microsatellite from X chromosome showed one of paternal origin and other of maternal origin. Microsatellite from 15 chromosome showed a deletion of D15S541, D15S11, GARB3, and D15S113 of maternal origin.

Molecular analysis using Multiple Ligation Probe Amplification methodology (SALSA MLPA probemix kit P245-A2, MRC-Holland, Amsterdam, NL) confirmed the deletion of the PWS/AS critical region, while the dose of control probes located at 15q24.1 was normal. In order to determine the origin of the chromosome with the 15q11.2-q13 deletion, we analyzed five microsatellite markers:* D15S541*,* D15S11,* and* GABR3*, located within the critical region of PWS/AS, and* D15S131* and* D15S984* located outside of the critical region, which were used as controls. Analysis of* D15S541*,* D15S11*, and* GABR3* markers confirmed the deletion of 15q11.2-q13 on the maternal chromosome (AS) (Figure 4). Control markers showed paternal and maternal 15 chromosome.

The paternal origin of the extra X chromosome was determined by analysis of a set of microsatellites located on the X chromosome by multiplex PCR (Figure 3).

## 3. Discussion

To our knowledge, this is the first case of a patient with coexisting AS and KS. The combined effects of both syndromes are not clear, since the patient is currently only 11 months old. The main phenotypic effects in KS are manifested in the mid-30s and in AS do not appear until 2-3 years after birth. This is one of the earliest diagnosed cases of AS, and it may be due to the presence of two syndromes. We can expect that the main phenotypic features that he will exhibit will be those of AS, since typical KS clinical features are much milder. We showed that the extra X chromosome was of paternal origin and the deletion in 15 chromosome was of maternal origin. Paternal and maternal sex chromosome nondisjunction contribute equally as causes of KS. Both parents were young and had normal karyotypes. Therefore, the abnormalities are most probably a coincidental event in our patient. In light of this, we estimate that the recurrence risk in the next pregnancy for both syndromes is low.

Early diagnosis is essential for optimal treatment of AS children. Abnormal EEG is used as diagnostic criteria since it is present in almost all AS patients [1, 14]. It has been suggested that EEG abnormalities are age-dependent: they usually appear early and decrease with age [15]. Seizures are a common feature observed in AS patients [1]. Seizures and EEG abnormalities were not detected in our patient, maybe because he is 11 months old and these do not usually occur until 22–24 months of age.

The most likely situation is that both conditions are coincidental. Therefore, to calculate risk, both conditions must be considered separately. Hence, KS patients with uncommon clinical features—such as the hypotonia, feeding difficulties, or frequent laughing observed in our patient—may be considered for assessment of another associated condition. Since there are no prenatal findings of AS and/or KS, for this couple we recommend offering cytogenetic prenatal diagnosis and FISH or array-CGH for AS.
